# Dental Plaster

**Published:** 1883-10

**Authors:** 


### DENTAL PLASTER.

If others have met with the same difficulty that we have in procuring fine plaster-of-paris for laboratory uses, they will be obliged to us for calling their attention to that manufactured by the Newark Lime and Cement Mfg. Co., of Newark, N. J. We have been using it for some time, and it is not only exceedingly fine, but it sets very hard. For taking impressions it is the best that we have ever met.

